# Conditioned Sounds Enhance Visual Processing

**DOI:** 10.1371/journal.pone.0106860

**Published:** 2014-09-05

**Authors:** Fabrizio Leo, Uta Noppeney

**Affiliations:** 1 Max Planck Institute for Biological Cybernetics, Tübingen, Germany; 2 Institute of Biological Psychology, Otto-von-Guericke University, Magdeburg, Germany; 3 Department of Robotics, Brain and Cognitive Sciences, Istituto Italiano di Tecnologia, Genova, Italy; 4 Computational Neuroscience and Cognitive Robotics Centre, University of Birmingham, Birmingham, United Kingdom; University of Sydney, Australia

## Abstract

This psychophysics study investigated whether prior auditory conditioning influences how a sound interacts with visual perception. In the conditioning phase, subjects were presented with three pure tones ( =  conditioned stimuli, CS) that were paired with positive, negative or neutral unconditioned stimuli. As unconditioned reinforcers we employed pictures (highly pleasant, unpleasant and neutral) or monetary outcomes (+50 euro cents, −50 cents, 0 cents). In the subsequent visual selective attention paradigm, subjects were presented with near-threshold Gabors displayed in their left or right hemifield. Critically, the Gabors were presented in synchrony with one of the conditioned sounds. Subjects discriminated whether the Gabors were presented in their left or right hemifields. Participants determined the location more accurately when the Gabors were presented in synchrony with positive relative to neutral sounds irrespective of reinforcer type. Thus, previously rewarded relative to neutral sounds increased the bottom-up salience of the visual Gabors. Our results are the first demonstration that prior auditory conditioning is a potent mechanism to modulate the effect of sounds on visual perception.

## Introduction

To form a coherent percept of the environment, the brain needs to integrate information from multiple sensory modalities. Critically, sensory signals should only be integrated, if they are generated by the same event as indicated by temporal, spatial or higher order structural (e.g. semantic) correspondences. In order to be integrated, sensory signals thus need to co-occur within a spatial and temporal window of integration and be structurally similar [1], [2], [3], [4].

Multisensory integration provides two important advantages for the survival of an organism. First, it enables an observer to estimate environmental properties such as spatial location more reliably. Second, it facilitates detection of events of interest. For instance, in a redundant target paradigm, participants are faster and more accurate when responding to multisensory relative to unisensory events. Likewise, in intersensory selective attention tasks, the detection of a visual target can be facilitated by the concurrent presentation of a sound as indicated by increased visual detection sensitivity d′ [5], [6] and greater subjective visual intensity [7] as well as shorter visual detection latencies [8].

Surprisingly, synchronous but otherwise uninformative sounds do not only facilitate stimulus detection, but also enhance the discrimination of visual patterns, orientation or motion direction [9], [10], [11]. An increase in discrimination performance may result from low level audiovisual integration mechanisms that increase stimulus salience. Alternatively, a concurrent sound may facilitate detection and discrimination of a perceptually weak signal in the visual modality by reducing an observer's temporal uncertainty about its occurrence. Indeed, previous psychophysical research has demonstrated that a consistent temporal relationship between the visual and auditory signals is critical for the sound-induced benefit to emerge in visual discrimination [12].

The current study investigates whether prior auditory conditioning influences the effect of concurrent sounds on visual discrimination. In the unisensory domain, it is well-established that classical auditory conditioning can induce plasticity in auditory cortices [13], [14], [15], [16], [17], [18]. In particular, auditory conditioning increased the representations of a conditioned relative to a neutral sound in primary auditory cortex demonstrating that primary sensory cortices also encode the behavioural relevance of a stimulus [19], [20], [21], [22]. However, as conditioning research has been limited to unisensory contexts, it remains unknown whether this conditioning-induced auditory plasticity also affects how sounds interact with visual processing.

To investigate whether prior auditory conditioning modulates audiovisual integration, we presented participants with visual stimuli at threshold intensity in a visual discrimination task. Critically, the visual signals were presented in synchrony with sounds that had previously been conditioned with a positive or negative reinforcer or associated with a neutral stimulus. In separate experiments, we employed money (gain, neutral, loss) or pictures (positive, neutral, negative valence) as unconditioned reinforcers in the prior conditioning phase. If prior conditioning affects audiovisual integration, we would expect enhanced visual discrimination accuracy and faster response times for visual stimuli that were presented together with previously rewarded/punished relative to neutral sounds.

## Materials and Methods

### Participants

Initially, our study included 24 participants (12 per group). Even though this study revealed significant effects of outcome valence on performance accuracy and reaction times, reviewers were concerned about insufficient power. Using Gpower [23] we therefore performed a power analysis assuming an effect size f = 0.2574 based on the initially observed main effect of outcome valence on performance accuracy. This power analysis demonstrated that at least 34 participants were required to detect this effect with a power (1- ß) of 0.9 and α = .05. Hence, thirty-six participants (mean age: 33.3 years; std: 8.4; 16 females) participated in the final and reported study. Importantly, this study replicated the initially reported effects.

All participants reported normal or corrected to normal visual acuity and normal hearing. All were naïve to the purpose of the study, were paid for their time and provided written informed consent to participate in the study. Both consent procedure and the study were approved by the ethics committee of the University of Tübingen.

### Overview of experimental design

This conditioning study investigated whether prior auditory conditioning modulates the effect of a sound on visual discrimination performance. During the initial conditioning phase, participants learnt to associate a particular sound with positive, neutral or negative outcomes. In two separate experiments, we employed pictures (highly pleasant, unpleasant and neutral IAPS) or monetary outcomes (+50 euro cents, −50 cents, 0 cents) as unconditioned reinforcers. Hence, the 3×2 factorial design manipulated: (i) valence of outcome: reward, neutral, punishment as a within subject factor and (ii) type of reinforcement: picture vs. money as a between subject factor.

After the conditioning phase, participants performed a visual discrimination task in an intersensory selective attention paradigm. Critically, the visual stimuli were presented in synchrony with one of the three sounds that had previously been paired with a positive, neutral or negative outcome. To avoid extinction, the visual discrimination task alternated with additional conditioning blocks.

In the following, we will describe the conditioning and the visual discrimination paradigms in greater detail.

### Conditioning phase

Participants were divided into two groups of eighteen participants each. Group 1 was exposed to money, group 2 to pictures as reinforcers. The initial conditioning phase was designed to establish an association between a particular sound frequency (CS, conditioned stimulus) and a specific outcome (US, unconditioned stimulus: reward, neutral or punishment).

### Monetary conditioning phase

#### Stimuli

Auditory stimuli were pure tones of 200 ms duration, sampled at 44.1 kHz. We employed three different tones that differed only in sound frequency: a 500 Hz, a 750 Hz and a 1000 Hz sound. All sounds had an intensity level of 70 dB SPL and 5 ms onset and offset ramps to avoid clicks. Before performing the experiment, we verified that participants of both groups were able to discriminate the three sound frequencies in 30 trials using a 3-alternative forced choice (3-AFC) frequency discrimination test. In particular, participants were asked to report whether the presented sound had a high, medium or low frequency by pressing the key ‘1’, ‘2’ or ‘3’ of the keyboard (average accuracy was 92.9%±1.1 SE).

#### Procedure

**Figure 1:**
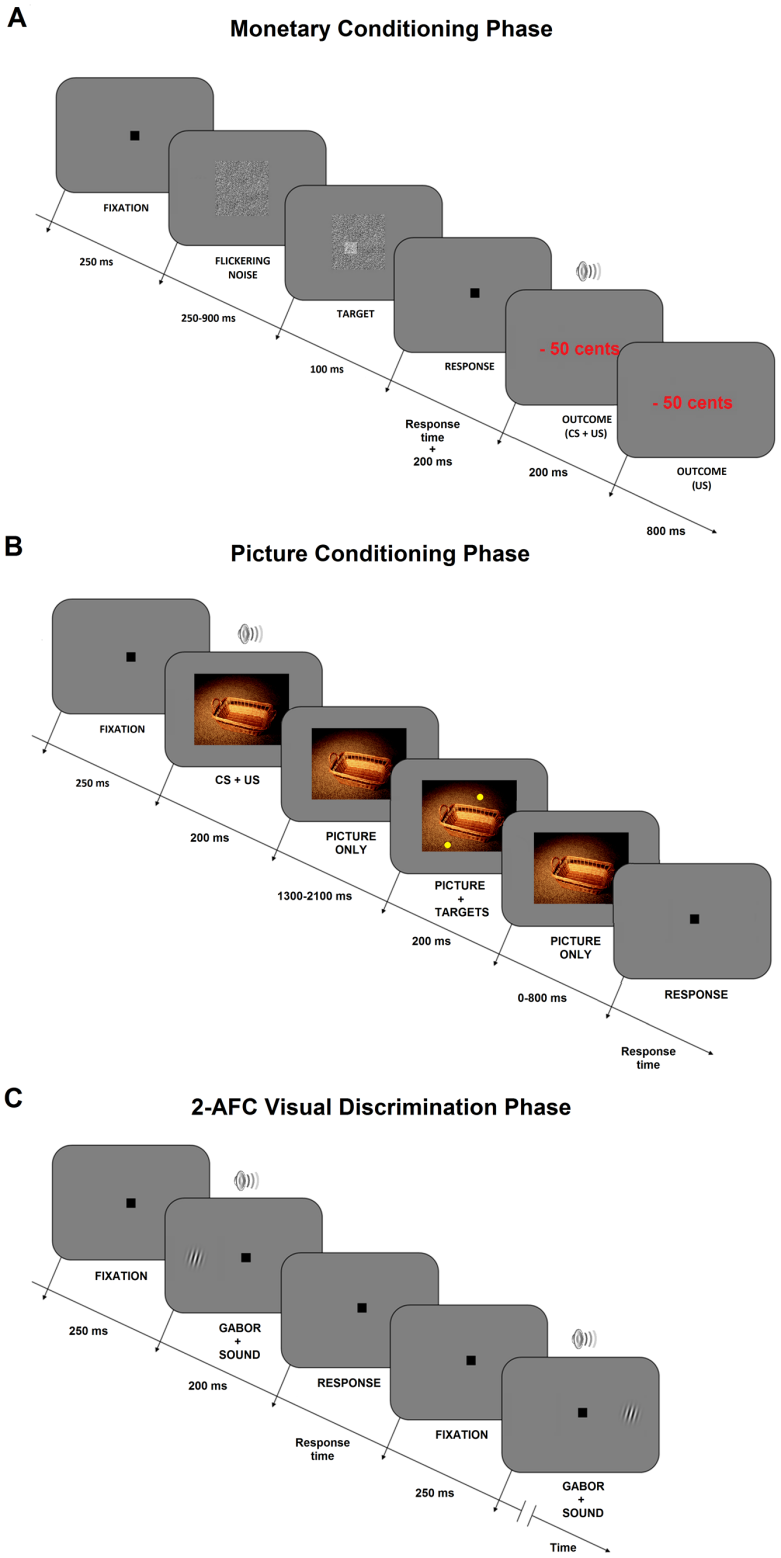
Example trials of the monetary and picture conditioning phase and the visual discrimination paradigm. (A) Monetary conditioning phase. On each trial, participants responded to the brief presentation of a small square within a noise square as quickly and accurately as possible. After each response, they received a monetary outcome that was positive, neutral or negative (e.g., −50 cents, in the Figure; US). The outcome was presented concurrently with a particular sound (CS). (B) Picture conditioning phase. On each trial, participants were presented with a picture (US) in synchrony with a particular sound (CS). After a variable temporal interval, two or three yellow dots were superimposed onto the picture. Participants reported the number of dots. (C) 2-AFC visual discrimination phase. On each trial, participants were presented with a near-threshold Gabor in synchrony with one of the conditioned sounds (CS). They discriminated whether the Gabor was presented in their left or right hemifield.

Participants were instructed to play a monetary game that was structured as follows. Each of the 60 trials (20 trials for each sound frequency) started with a 13°×13° centered square composed of dynamic visual Gaussian noise with a mean luminance of the midgray background of 65 cd/m^2^. The noise rectangle (check size = 1.5 arcmin) was generated for each frame at a refresh rate of 60 Hz, by sampling the intensity values for each pixel following a Gaussian distribution with a standard deviation of 0.2 centered around the mean luminance value. The duration of the visual flickering noise was 1 s. During this time, a dot (1.3° of diameter) composed of Gaussian noise with a greater standard deviation (0.35) was superimposed onto the background flickering noise for 100 ms (see panel A of Figure 1). The dot location was sampled from a uniform two-dimensional distribution within the visual angle of 11°×11°. The onset time was sampled from a uniform distribution between 250 and 900 ms after the onset of the noise square.

Participants were asked to detect the dot onset as quickly as possible by pressing the left arrow of the keyboard. Participants were informed that they would win 50 real Euro cents for fast responses, lose 50 cents for slow responses and receive neutral outcome for intermediately fast responses. However, unknown to the subject the outcome of the monetary reward on each trial was randomized and hence independent from the speed or accuracy of participants' response. In this way, we ensured that the number of trials was identical for each possible association between outcome and tone across all different conditions. At the end of the experiment, participants were informed that they obtained equal number of gains and losses and were not paid additional money. 200 ms after pressing the response button, the reward outcome was presented on the screen for 1 s (US: words written in red: ‘+50 cents’, ‘−50 cents’ or ‘±00 cents’) and a tone of a particular frequency was played concurrently for 200 ms (CS; see Stimuli section). The onset of the presentation of the reward outcome and the tone was synchronous. For each participant, a particular monetary outcome was consistently paired with a specific sound frequency (e.g. monetary reward with a 1000 Hz sound, monetary punishment with a 500 Hz sound, neutral outcome with a 750 Hz sound). The associations between US and CS were counterbalanced across participants such that each sound frequency was equally often paired with each monetary outcome (i.e., 18 participants divided by 6 possible associations between US and CS resulting in 3 participants per sound-outcome pairing).

### Picture conditioning phase

#### Stimuli

Auditory stimuli were identical to the pure tones described in the Stimuli section of the Monetary conditioning phase.

Thirty pictures from the IAPS International Affective Picture System [24] were selected based on their normative ratings of hedonic valence and emotional arousal as listed in the IAPS manual. Pleasant and unpleasant pictures (US) were selected independently for each gender in order to obtain the highest values of valence and arousal. The 10 pleasant pictures had mainly sexual content or represented adventures (after pooling for gender; mean valence: 7.8, SD: 0.25; mean arousal: 6.85, SD: 0.33). The 10 unpleasant pictures included mutilated bodies, attack scenes and disgusting objects (mean valence: 2.15, SD: 0.2; mean arousal: 6.85, SD: 0.41). The 10 neutral pictures served as control stimuli and included mainly landscapes, people and objects (mean valence: 4.9, SD: 0.28; mean arousal: 2.4, SD: 0.19). Pleasant and unpleasant pictures were selected to be matched in terms of arousal and the degree to which their valence deviated from the neutral pictures (i.e. the absolute differences between (i) pleasant minus neutral picture and (i) unpleasant minus neutral pictures was not significantly different: 2.9 and 2.75, respectively; *p* = .12).

#### Procedure

Participants were presented with pleasant, neutral and unpleasant pictures in a randomized fashion (horizontal visual angle: 26.3°, vertical visual angle: 19.7°). The pictures were displayed in the centre of the screen. Critically, the onset of each picture was in synchrony with the presentation of a sound of a particular frequency (total picture presentation duration: 2.5 s; sound duration: 200 ms). As in the monetary conditioning phase, each sound frequency was consistently paired with pictures of positive, neutral or negative valence. Likewise, the associations were counterbalanced across participants between US and CS as in the monetary conditioning phase.

Each trial started with 250 ms fixation, followed by the concurrent onset of a picture and a sound. After a variable time period between 1.5 and 2.3 s, two or three yellow dots (diameter: 1.3°; RGB values: [1 1 0]) appeared for 0.2 s at random locations on the picture. Participants were asked to report how many dots were presented by pressing either the key number ‘2’ or the key number ‘3’. This visual task was employed to ensure that participants attended the pictures. Upon participant's response, the next trial was started.

### Two-alternative forced choice (2-AFC) visual discrimination phase

#### Stimuli

Visual target stimuli were vertically oriented Gabor patches with spatial frequency of 3 cycles per degree. They were presented at 75% of correct contrast detection level, as estimated individually for each participant and for each visual hemifield using a staircase QUEST procedure.

#### Procedure

On each trial, a Gabor patch was presented at 6° or −6° eccentricity for 200 ms. Critically, the Gabor patch was presented in synchrony with a sound of 1000 Hz, 750 Hz or 500 Hz (duration 200 ms, see panel B of Figure 1). These sounds had been paired with positive, neutral or negative outcomes in the prior conditioning phase. Hence, the experimental paradigm included three conditions: (i) rewarded AV, (ii) neutral AV or (iii) punished AV.

Participants were instructed to fixate a black square in the centre of the screen and to discriminate whether the Gabor patch was presented in their left or right hemifield as accurately as possible (speed was less stressed). Please note that while this task is formally a discrimination task, it will involve detection processes of the stimulus in one of the two hemifields.

To avoid extinction, blocks of 2-AFC task alternated with additional conditioning blocks that reinforced the association between sound and monetary or picture outcome (see above). There were nine 2AFC blocks per participant. Each 2-AFC block included 36 trials resulting in 324 trials in total (i.e. 108 2-AFC trials for each outcome). Each conditioning block included 30 trials.

## Results

We evaluated the effect of prior auditory conditioning in terms of performance accuracy and response times on the visual discrimination task, where participants indicated whether a Gabor patch was presented in their left or right hemifield. As performance accuracy was comparable for left and right visual targets (discrimination accuracy for targets presented in the left hemifield: 84%±2 SE; targets in the right hemifield: 83%±2, t(35) = .67, *p* = .50). Hence, we pooled the responses over targets presented in the two visual hemifields.

For each participant, we computed performance accuracy (i.e. percentage correct) and response times (RT). Our central question was whether the effect of a sound on visual discrimination performance can be influenced by prior conditioning in terms of (i) outcome valence or (ii) type of conditioning/reinforcer. Hence, both performance accuracy and response times were analyzed in separate 3 (sound outcome valence: rewarded, neutral, punished) ×2 (reinforcer type: money vs. picture) repeated measure ANOVAs. The ANOVA results are reported Greenhouse-Geisser corrected for non-sphericity (if required).

**Figure 2:**
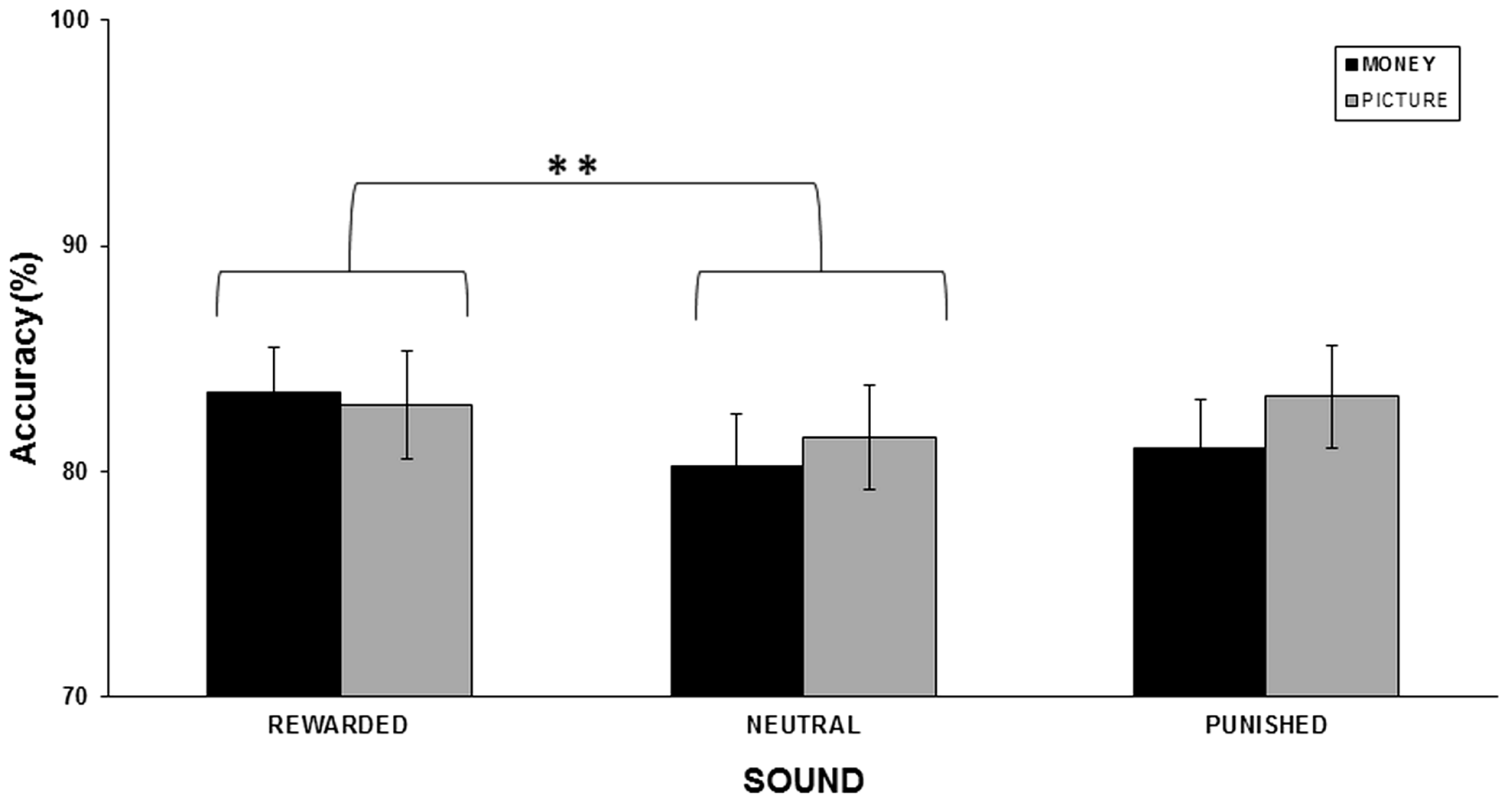
Performance accuracy in the visual discrimination phase. Across participants' mean performance accuracy (SEM indicated) for each condition in the visual discrimination task for the monetary (black) and picture conditioning paradigm (gray). Asterisks indicate significant differences between rewarded and neutral sound condition, * *p*<0.01. See main text.

For performance accuracy (see Figure 2), the repeated measure ANOVA identified a significant main effect of sound outcome valence [*F*(1.76,29.92) = 5.65; *p* = .01], but no significant main effect of reinforcer type [*F*(1,17) = .21; *p* = .65] and no interaction [*F*(1.98,33.74) = 2.4; *p* = .10]. Follow-up Newman-Keuls tests revealed that the visual discrimination accuracy was significantly higher for the rewarded AV condition (accuracy  = 83%±1.6) than for the neutral AV condition (80.7±1.7; *p* = .005). Furthermore, there was also a marginal (but nearly significant) trend toward enhanced discrimination accuracy for punished sounds (accuracy  = 82±1.7) as compared to neutral sounds (*p* = .09; see Table 1).

**Table 1:** Mean RTs and Accuracy.

Money			Picture		
Sound Outcome Valence	Accuracy	RT	Sound Outcome Valence	Accuracy	RT
**Rewarded**	83 (8.7)	643 (165)	**Rewarded**	82.9 (10.1)	613 (162)
**Neutral**	80 (10)	670 (170)	**Neutral**	81.5 (9.9)	606 (146)
**Punished**	80.7 (9)	640 (152)	**Punished**	83.3 (9.7)	623 (173)

Across participants' mean accuracy (%) and response time (ms) for each sound outcome valence, separately for the monetary and the picture conditioning paradigms. Standard deviations are reported in brackets.

**Figure 3:**
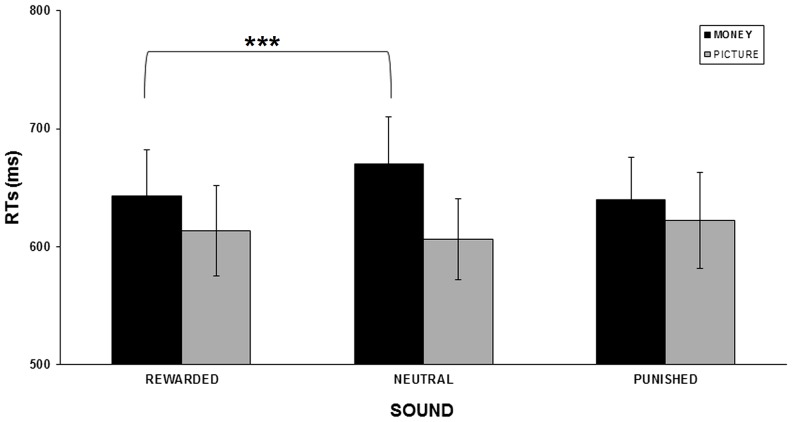
Response times in the visual discrimination phase. Across participants' mean response times (SEM indicated) for each condition in the visual discrimination task for the monetary (black) and picture conditioning paradigm (gray). Asterisks indicate significant differences between rewarded and neutral sound conditions in the monetary group, ** p<0.001. See main text.

By contrast, for response times (see Figure 3), the repeated measure ANOVA identified a significant interaction between reinforcer type and sound outcome valence [*F*(1.31,22.31) = 10.93; *p* = .001], but no significant main effects. To further characterize the interaction, we tested for the simple main effects using paired two-tailed t-tests. In other words, we compared rewarded with neutral sounds and punished with neutral sounds, separately for each reinforcer type. P-values are reported after Bonferroni's correction for multiple comparisons.

We observed faster responses for rewarded (RT = 643 ms±49) as compared to neutral sounds (RT = 670 ms±50) in the monetary conditioning group (*p* = .0008). All the other comparisons were not significant (all *p* values >.07; see Table 1).

## Discussion

Accumulating evidence from rodents, non-human primates and human neuroimaging studies has documented experience-dependent plasticity in auditory cortex [25], [26], [27]. In particular, conditioning has proven an effective approach for modifying stimulus representations in auditory cortex. For instance, human neuroimaging studies revealed increased neural responses in auditory cortex during associative learning [14] and enhanced frequency specific responses to tones after conditioning [13], [15], [17]. Collectively, these studies have demonstrated that representations in primary auditory cortex flexibly encode the behavioural relevance of the auditory stimuli. This raises the question whether the impact of sounds on visual processing is also influenced by their behavioural relevance as acquired through prior conditioning history and/or task-context. In other words, does prior conditioning affect how sounds interact with vision?

Numerous previous studies have demonstrated that audiovisual interactions depend on physical stimulus characteristics such as signal strength [28] or stimulus complexity [29]. Likewise, audiovisual interactions of naturalistic meaningful stimuli such as speech or object sounds depend on higher order semantic congruency [30], [31] or speech intelligibility [32], [33], [34], [35]. However, to our knowledge, this is the first study to investigate whether behavioural relevance as acquired through prior conditioning changes audiovisual interactions of simple stimuli. In particular, we asked whether prior auditory conditioning changes the impact of sounds on visual discrimination performance.

To address this question, participants learnt to associate three different tones with positive, neutral or negative outcomes using a monetary or picture conditioning paradigm. In the subsequent experimental phase, participants had to discriminate whether a near-threshold Gabor patch was presented in their left or right hemifield. Critically, the Gabor patch was presented in synchrony with a central tone that had previously been paired with positive, neutral or negative outcomes. Our results demonstrate that previously rewarded tones increase performance accuracy on this visual discrimination task relative to neutral tones irrespective of whether money or pictures were employed as reinforcers. These results demonstrate that indeed the behavioural relevance of sensory signals as acquired during prior conditioning flexibly determines audiovisual interactions. The effect of a concurrent simple tone on visual discrimination is amplified, if it signals a rewarding outcome. These results suggest that the plastic changes previously observed in primary auditory cortex for auditory conditioning do not only affect auditory processing, but transfer to the visual processing stream. Thus, prior conditioning may enhance the salience of the sound possibly via plastic changes in primary auditory cortices. This increase in salience activates higher order attentional systems that are shared across sensory modalities leading to increased visual discrimination performance [36], [37], [38]. Critically, our results demonstrate that the rapid conditioning-induced plasticity in primary auditory areas that has been shown in previous neuroimaging studies does not only induce ‘local’ effects on auditory processing, but transfers to other sensory modalities.

While an increase in performance was predominantly found for previously rewarded sounds, a similar though non-significant trend was observed also for tones that had previously been paired with negative outcomes. Only few previous studies have directly compared reward and punishment in the same experimental paradigm in animals or humans [39]. Previous studies in insects have demonstrated that punishment memory decayed more rapidly than reward memory in olfactory learning in crickets [40] and fruit-flies [41], [42] and in visual pattern learning in crickets [43]. This effect has been proposed to be a direct consequence of the different neurotransmitters involved in reward and punishment learning, that is octopamine (invertebrate counterpart of noradrenaline) and dopamine, respectively [40], [43].

In our specific study, the reward primacy may be related to the fact that winning or losing 50 cents is asymmetrical from a neuroeconomical perspective. However, this hypothesis contradicts previous findings demonstrating an increase in skin conductance, pupil dilation and heart rate in response to monetary loss as compared to gain [44], [45], suggesting the possibility that losing a particular amount of money would be experienced more strongly than gaining the same amount. As the difference between tones that had previously been paired with negative or positive outcomes was not significant, it is premature to draw firm conclusions as to whether valence critically modulates auditory effects on visual discrimination.

While the conditioning effects on performance accuracy did not depend on reinforcer type, we observed a significant interaction between reinforcer type and conditioning history for responses times. More specifically, we observed faster response times for rewarded relative to neutral sounds in the visual discrimination task only for the monetary conditioning paradigm. This difference may relate to the particular task-constraints of the conditioning phases for the picture and monetary reinforcer types. Thus, monetary conditioning was applied in a game context where participants were instructed to respond as fast as possible to visual targets. Moreover, participants were told that they would be rewarded for fast responses, but punished for slow responses. Given the vast literature on top-down task-related factors in auditory plasticity, these contextual factors from the conditioning phase are likely to co-determine the effects of conditioned tones on visual processing.

In conclusion, our results demonstrate that prior conditioning and even the particular conditioning paradigm affect the effect of simple tones on visual processing in a visual discrimination paradigm. Simple tones that have gained behavioral relevance by nature of being rewarded during the conditioning phase are more potent in increasing the salience of the visual signal and facilitating visual perception. Future neuroimaging studies are needed to define the neural mechanisms underlying these audiovisual benefits that depend on prior conditioning. For instance, the enhanced representations in primary auditory cortex for previously rewarded tones may directly influence and enhance signal salience in primary visual cortices [46], [47]. Alternatively, previously rewarded relative to neutral tones may activate generic attentional resources in higher order frontoparietal areas [48]. Finally, the audiovisual interactions may be mediated via the superior colliculus, a subcortical structure that is particularly rich in multisensory neurons and has previously been implicated in audiovisual benefits in simple detection tasks [49], [50], [51], [52].
